# Thirty Years of Mungbean Genome Research: Where Do We Stand and What Have We Learned?

**DOI:** 10.3389/fpls.2022.944721

**Published:** 2022-07-15

**Authors:** Prakit Somta, Kularb Laosatit, Xingxing Yuan, Xin Chen

**Affiliations:** ^1^Department of Agronomy, Faculty of Agriculture at Kamphaeng Saen, Kasetsart University, Nakhon Pathom, Thailand; ^2^Institute of Industrial Crops, Jiangsu Academy of Agricultural Sciences, Nanjing, China

**Keywords:** mungbean, gene mapping, genome research, genomics, QTL, marker-assisted breeding

## Abstract

Mungbean is a socioeconomically important legume crop in Asia that is currently in high demand by consumers and industries both as dried beans and in plant-based protein foods. Marker-assisted and genomics-assisted breeding are promising approaches to efficiently and rapidly develop new cultivars with improved yield, quality, and resistance to biotic and abiotic stresses. Although mungbean was at the forefront of research at the dawn of the plant genomics era 30 years ago, the crop is a “slow runner” in genome research due to limited genomic resources, especially DNA markers. Significant progress in mungbean genome research was achieved only within the last 10 years, notably after the release of the VC1973A draft reference genome constructed using next-generation sequencing technology, which enabled fast and efficient DNA marker development, gene mapping, and identification of candidate genes for complex traits. Resistance to biotic stresses has dominated mungbean genome research to date; however, research is on the rise. In this study, we provide an overview of the past progress and current status of mungbean genomics research. We also discuss and evaluate some research results to provide a better understanding of mungbean genomics.

## Introduction

Mungbean [*V. radiata* (L.) R. Wilczek var. *radiata*] is an ancient legume crop from Asia. The crop is believed to have been domesticated from its wild form, *V. radiata* var. *sublobata* (Roxb.) Vercourt, in India about 4,000–4,500 years ago (Fuller and Harvey, 2006). Mungbean has been cultivated and consumed as a common food in China and Thailand for more than 2,000 years (Castillo, 2013; Tang et al., 2014). It is well-known for its detoxification activity and is used to refresh the mind, alleviate heat stroke, and reduce swelling in summer (Tang et al., 2014). Mungbean adapts well to various cropping systems due to its rapid growth, early maturity (60–75 days), relative tolerance to drought, and ability to improve soil fertility through atmospheric nitrogen (*N*_2_) fixation in symbiosis with *Rhizobium* and *Bradyrhizobium* bacteria in the soil (Yimram et al., 2009). Hence, it plays an important role in crop diversification and sustainable agricultural intensification in Asia.

Mungbean seeds are a source of protein, amino acids, carbohydrates, vitamins, and minerals. The seeds contain about 20–30% protein and 60–70% carbohydrate. Mungbean is an important protein source for people in cereal-based societies, especially in South Asia. Mungbean seeds are cooked and consumed in a variety of ways, and mungbean flour is used to prepare several kinds of food. The seeds are also processed into sprouts, snacks, pastes, starches, noodles, protein isolates, and protein concentrates (Nair and Schreinemachers, 2020). There is a growing interest in mungbean consumption and use because of its high protein content, high-quality starch, and other nutritional contents. Mungbean seeds have become a major material used in the production of plant-based protein foods, such as egg and meat substitutes.

Although official statistical data on the global production area and seed yield of mungbean are not available, the production area is about 7.5–8.0 million ha, about 80–90% of which is in Asia (Nair and Schreinemachers, 2020; Anonymous, 2021). Mungbean production areas outside Asia, including in Australia, America, and Africa, are increasing. This is driven by increasing consumer demand for dry legumes and plant-based protein foods. The biggest producer and consumer of mungbean is India, with about 4.5 million ha cultivated and a total production of 2.5 million tons (Anonymous, 2021). Myanmar is the second-highest producer and largest exporter, with about 1.2 million ha cultivated and a total production of 1.5 million tons (MAOLI, 2019). Although mungbean production areas are increasing, the yield is low, at only about 115 kg/ha (Nair and Schreinemachers, 2020), and production is challenged by insect pests, diseases, and unsuitable environments (Pandey et al., 2018; Nair et al., 2019). Insects and diseases are mainly controlled by applying pesticides, which increases production costs and is hazardous to farmers, consumers, and the environment. There is a need to develop new mungbean cultivars that fulfill the needs of farmers, consumers, and processors.

**Figure 1:**
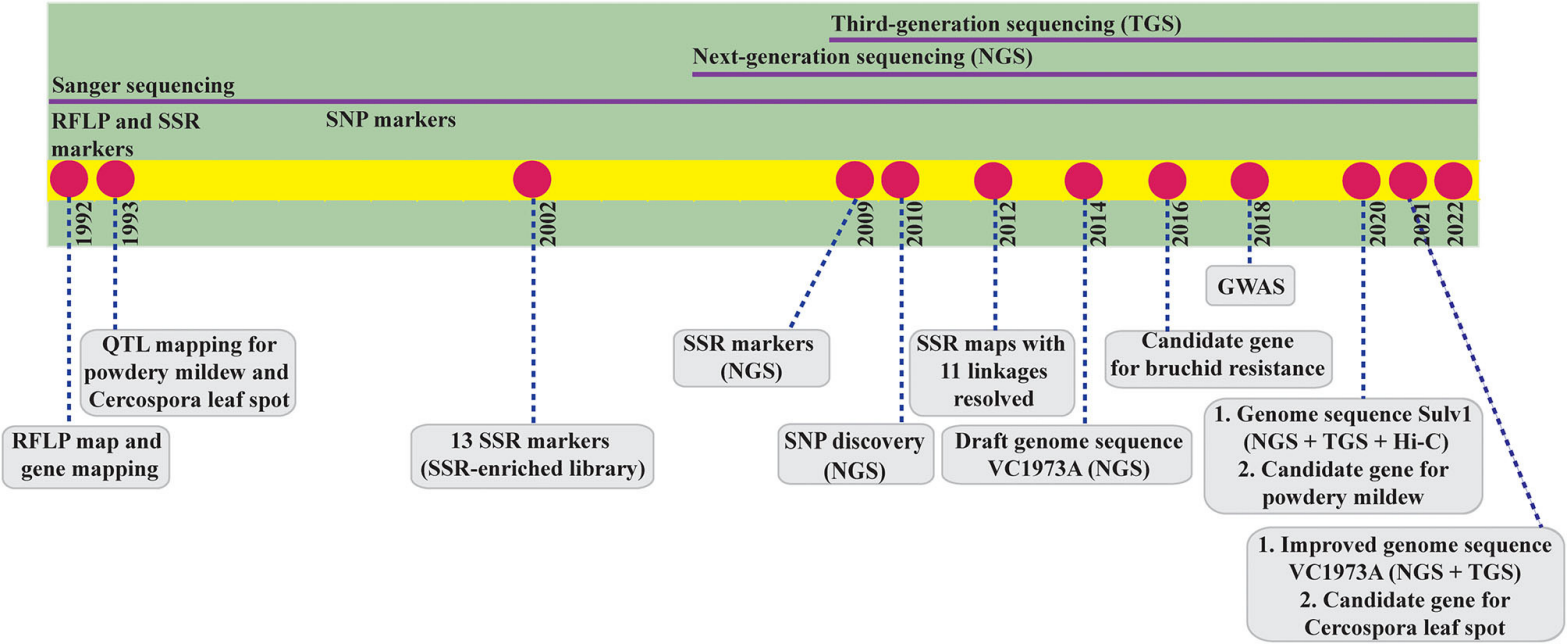
Timeline of mungbean genome research since the first publication of genome mapping in 1992. RFLP, restriction fragment length polymorphism; QTL, quantitative trait locus; SSR, simple sequence repeat; SNP, single nucleotide polymorphism; GWAS, genome-wide association study; Hi-C, high-throughput chromosome conformation capture.

Despite its high socioeconomic importance, mungbean is neglected and underfunded in breeding research at both the national and international level, particularly in the field of genomics, which holds great promise for modern plant breeding. Mungbean is an ideal crop for genomics study because it is self-pollinating and diploid (2n = 2x = 22) and has a small genome size of 493.6–579.0 megabase (Mb) pairs (Arumuganathan and Earle, 1991; Kang et al., 2014; Liu M. S. et al., 2016) and a short life cycle with early maturity (60–75 days). In fact, mungbean was among the first legumes subjected to genomics research, with the first genome mapping reported 30 years ago, but today, advances in mungbean genomics lag behind other legume crops with similar importance (Figure 1).

The most recent review on mungbean genomics was published 7 years ago (Kim et al., 2015). In that review, the authors mainly described genomic and genetic resources for the genetic improvement of mungbean. In addition, rapid and extensive progress has been made in mungbean genome research. In this study, we review all aspects of mungbean genomics research, past and present, and discuss and evaluate some research results to provide in-depth, wide-ranging, and recent analysis of mungbean genome research.

## A Brief History of Mungbean Breeding

Early mungbean genetics and breeding research occurred in India, the Philippines, and the USA. In India, germplasm collection and evaluation began in the 1920s, and pure lines were isolated (Bose, 1932). The first mungbean cultivar released for farmers was “Type 1” in 1936. The first cultivar developed through hybridization and selection and released to farmers was “Type 44,” and this was released in 1948. All cultivars used in this period were small-seeded, with non-uniform maturity, photoperiod sensitivity, and low yield. The average yield was only about 0.4–0.8 ton/ha. Since 1980s, many cultivars with improved yield, large seed, uniform maturity, non-sensitivity to photoperiod, and resistance to diseases have been developed.

In the Philippines, various landraces and introduced germplasms were tested before 1916 and the breeding programs began in 1933 (Catipon et al., 1988). Landraces were collected and pure lines were isolated, selected, and evaluated, resulting in six pure lines released to farmers and becoming very popular (Ballon et al., 1977). In 1956, a national breeding program was set up to develop cultivars with high yield that were semi-dwarf, with uniform maturity, large and shiny seed, non-shattering pods, and high seed protein and carbohydrate content by hybridization and selection, resulting in the release of cultivars “MG50-10A” and “MD15-2” in 1969 (Catipon et al., 1988). These cultivars yielded about 1.2 tons/ha, about double that of the pure-line cultivars. The improved cultivars revolutionized the yield, plant architecture, and phenology of mungbean, although they were still susceptible to diseases. In the mid-1970s, cultivars with high yield, good architecture, and disease resistance were successfully developed using resistance germplasm from India (Catipon et al., 1988).

In the USA, before 1914, the US Department of Agriculture (USDA) introduced and evaluated mungbean germplasm from several countries. Three cultivars, “Berken,” “Oklahoma 12,” and “Kiloga,” developed by selection from introduced germplasm, were released in 1963 (Matlock and Oswalt, 1963). In the early 1970s, the International Mungbean Nursery (IMN) was organized by the University of Missouri and later coordinated by the Asian Vegetable Research and Development Center in Taiwan [now the World Vegetable Center (WorldVeg)]. The IMN stimulated international cooperation in mungbean breeding.

Since WorldVeg was established in 1971, the center has played a key role in mungbean germplasm collection, characterization, and breeding. By exploiting the high yield and good agronomic traits of improved cultivars/lines from the Philippines and the disease resistance of Indian germplasm as parents, newly improved lines with better yield, agronomic traits, and disease resistance were bred (Fernandez and Shanmugasundaram, 1988). The improved lines yielded up to 1.8–2.2 tons/ha and boosted mungbean cultivation and production in Asia from the 1980s to 2000s. WorldVeg mungbean lines were also used as parents in breeding programs in several countries. The adoption and use of the WorldVeg breeding lines have transformed the mungbean from a marginal crop to a major crop in Asia. For example, the cultivars developed and released by the WorldVeg generated economic gains of USD 1.4 billion in Myanmar from 1980 to 2016 (Sequeros et al., 2020).

## Mungbean at the Dawn of Plant Genomics Era

One of the goals of plant genomics research is to utilize tools, knowledge, and information generated from research to improve crops. Plant genomics research relies on the availability of polymorphic DNA markers and dense genetic linkages. The era of crop genomics began in the late 1980s with the development of restriction fragment length polymorphism (RFLP) markers and genetic linkage maps of several economic crops, including rice (McCouch et al., 1988), tomato (Bernatzky and Tanksley, 1986), and maize (Helentjaris, 1987). The publication of quantitative trait loci (QTL) mapping of tomato in 1988 (Paterson et al., 1988) stimulated gene mapping of crop plants. Genome research in legume crops began in the late 1980s to early 1990s. The first genome mapping of mungbean was published in 1992 (Fatokun et al., 1992; Young et al., 1992), and the first genetic linkage map of mungbean was officially published in 1993 (Menancio-Hautea et al., 1993). Genetic linkage maps of some important legume crops, such as soybean [*Glycine max* (L.) Merr.] (Keim et al., 1990), common bean (*Phaseolus vulgaris* L.) (Vallejos et al., 1992), and cowpea [*Vigna unguiculata* (L.) Walp.] (Fatokun et al., 1993), were developed at around the same time. The mungbean linkage map was based solely on RFLP markers and was used to identify QTLs controlling seed size (Fatokun et al., 1992), insect resistance (Young et al., 1992), and disease resistance (Young et al., 1993). Comparative linkage analyses between mungbean and cowpea, common bean, and soybean were also published (Fatokun et al., 1992; Boutin et al., 1995), revealing high conservation of synteny between mungbean and these legumes. The orthologous seed weight QTLs between mungbean and cowpea (Fatokun et al., 1992) was reported in the first comparative genome analysis in legumes. However, after this period, mungbean genome research progressed very slowly.

Prior to 2010, only seven genetic linkage maps of mungbean were constructed and all of them were principally based on RFLP markers, which did not resolve the 11 haploid chromosomes of the mungbean (Menancio-Hautea et al., 1993; Boutin et al., 1995; Lambrides et al., 2000; Chaitieng et al., 2002; Humphry et al., 2002; Zhao et al., 2010). The RFLP probes utilized in those studies were mainly from other legumes, including common bean, cowpea, soybean, and lablab [*Lablab purpureus* (L.) Sweet]. In addition, gene mapping of mungbean involved only six traits, including seed size, seed dormancy, bruchid, bean bug (*Riptortus clavatus* Thunb.), and powdery mildew resistance (Kaga and Ishimoto, 1998; Chaitieng et al., 2002; Humphry et al., 2003, 2005; Mei et al., 2009; Hong et al., 2015). The slow progress was mainly due to the lack of efficient genomic resources, especially genetic markers (the most important tool in genomics study), such as simple sequence repeat (SSR), which was the most efficient DNA marker system at that time, even though the first report on SSR variation in mungbean was in the late 1990s (Yu et al., 1999). Fewer than 150 polymorphic SSR markers were reported for mungbean during this period (Kumar et al., 2002a,b; Gwag et al., 2006; Somta P. et al., 2008; Seehalak et al., 2009; Somta et al., 2009; Tangphatsornruang et al., 2009).

Since 2010, relatively rapid progress in mungbean genome research has been made. Several SSR-based maps were constructed and utilized for QTL mapping of various traits, including agronomic and domestication-related traits, biotic and abiotic stress resistance, and seed chemical contents (Kasettranan et al., 2010; Zhao et al., 2010; Chankaew et al., 2011, 2013; Isemura et al., 2012; Kajonphol et al., 2012; Prathet et al., 2012; Sompong et al., 2012; Chen et al., 2013; Kitsanachandee et al., 2013; Alam et al., 2014a,b; Wang et al., 2016; Liu et al., 2017; Masari et al., 2017). However, all of those maps were low-resolution, with nearly all of them containing <100 polymorphic markers. Highly polymorphic SSR markers from azuki bean [*Vigna angularis* (Willd.) Ohwi and Ohashi] (Wang et al., 2004) comprised the core of these maps. The first linkage maps that resolved the 11 haploid chromosomes of mungbean were published by Isemura et al. (2012) and Kajonphol et al. (2012), which were constructed from SSR markers of various legumes. The map developed by Isemura et al. (2012) was the densest, with 430 SSR markers and an average marker density of 1.78 centimorgans.

## Advanced Sequencing Technologies Accelerated Mungbean Genome Research

Next-generation sequencing (NGS) is a technology that involves the sequencing of millions of small fragments of DNA in parallel and enables rapid, inexpensive, and comprehensive analysis of the genomes of individual organisms and complex populations. NGS has revolutionized genome research on all organisms and in all disciplines. There are several NGS platforms, including Illumina/Solexa, Roche 454, Ion Torrent, and SOLiD sequencing. These platforms became available in the late 2000s and early 2010s, and they differ in terms of template preparation, sequencing chemistry, read length, and outputs per run (Metzter, 2010). In the early stage of NGS, parts of nuclear genomes and transcriptomes of mungbean were sequenced, which led to the identification of a massive number of SSRs and single nucleotide polymorphisms (SNPs) and the development of new SSR markers (Tangphatsornruang et al., 2009; Moe et al., 2011; Van et al., 2013; Gupta et al., 2014). NGS is also a powerful tool in the discovery and genotyping of large numbers of SNPs at a drastically reduced expense, such as genotyping-by-sequencing (GBS) (Elshire et al., 2011), which allowed highly dense genetic linkage maps to be developed for mungbean (Kang et al., 2014; Hwang et al., 2017; Mathivathana et al., 2019; Ha et al., 2021). In addition, the chloroplast genome of mungbean was fully sequenced by NGS (Tangphatsornruang et al., 2010).

The greatest contribution of NGS to mungbean genomics is whole-genome sequencing (WGS). A draft reference genome of the elite mungbean breeding line “VC1973” from WorldVeg was constructed on the chromosome level using Illumina/Solexa and Roche 454 sequencing (Kang et al., 2014). A 431-megabase (Mb) part (80%) of the total estimated mungbean genome (579 Mb) was sequenced and generated into 2,748 scaffolds with an N50 length of 1.62 Mb. Based on a linkage map of 1,321 SNP markers, scaffolds were assembled into 11 pseudochromosomes covering 314 Mb, equivalent to 73% of the assembled sequence. In total, only 22,427 protein-coding genes were identified, of which 18,378 genes were located on the pseudochromosomes. In addition to “VC1973A,” the whole genome of the wild mungbean accession “TC1966” was also sequenced (423 Mb, corresponding to about 84% of the estimated genome size). By comparing the genome sequences of mungbean “VC1973A,” “V2984,” and “TC1966,” millions of SNPs and hundreds of thousands of indels were discovered (Kang et al., 2014). Draft genome sequences, SNPs, and indels are highly useful resources for gene mapping and mining. A few years after the release of the draft genome sequence of “VC1973A,” the WGS of the breeding line “RIL59” was reported (Liu M. S. et al., 2016). A 455.2-Mb portion (88%) of the genome of RIL59 (517.1 Mb) was sequenced using Illumina sequencing and assembled into 2,509 scaffolds with an N50 of 676.7 kb and an average sequence length 181.4 Mb. A total of 36,939 protein-coding genes were predicted.

A complete and high-quality reference genome sequence serves as the basis for advanced genomic analysis, providing an opportunity to systemically identify and characterize the functions of genes. Third-generation sequencing, such as single-molecule real-time (SMRT) sequencing and nanopore sequencing, generates sequence reads with unprecedented lengths in less time and with more accuracy, but at a lower cost. Such technology is highly useful for the construction of contiguous and chromosome-scale genome sequences (Amarasinghe et al., 2020). Recently, a high-quality chromosome-scale genome assembly of the mungbean cultivar “Sulv1” was constructed using a combination of second-generation sequencing, third-generation sequencing, and Hi-C analysis (Yan et al., 2020). In the Sulv1 genome assembly, 473.7 Mb, covering 87.8% of the estimated genome size, was assembled and 99.32% of sequences were assigned to 11 pseudochromosomes, with scaffold N50 of 42.4 Mb and 33,924 predicted protein-coding genes. More recently, the genome sequence of VC1973 was improved using SMRT sequencing (Ha et al., 2021). The new genome assembly covered the total sequence of 476 Mb from 557 scaffolds with an N50 length of 5.2 Mb and only a 0.4% gap. The sequence corresponded to 87.5% of the VC1973A genome, oriented into 11 pseudochromosomes, and contained 30,958 genes. The new assembly was compared with the previous ones by mapping common SNP markers to both genome assemblies, which revealed many disagreements between the two assemblies. Nonetheless, comparative genome analysis of the new genome assembly and genomes of legumes closely related to mungbean demonstrated that the new mungbean assembly was more reliable that the previous one. The large difference in the number of identified genes in the genomes of VC1973A, RIL59, and Sulv1 suggests a substantial genome variation in mungbean.

Having more than one reference genome for mungbean is an advantage when analyzing gene discovery and genome structural variations (e.g., presence–absence variation, copy number variation, and chromosomal rearrangements) because different genomes of a species vary in both gene content and repetitive portions of the genome (Della Coletta et al., 2021). To date, the improved genome sequence of VC1973A and the genome sequences of Sulv1 and wild mungbean TC1966 have not been released to the public. Moreover, a large portion (12%) of VC1973A, RIL59, and Sulv1 genome sequences is still not covered by WGS. Additional genome sequencing is necessary to increase genome coverage as much as possible. The puzzle of mungbean genome variation may be resolved through pangenome construction. Additional reference genome sequences of mungbean are being constructed and a mungbean pangenome project has been initiated (Nair et al., 2022).

The NGS technology also provides access to genome-wide transcript variation for understanding the functional elements of the genome, molecular constituents of cells and tissues, and phenotypic variations *via* transcriptome sequencing (Wang et al., 2009). Before 2000, there was only one transcriptomics study on mungbean (Chen et al., 2008). When the cost of NGS became affordable around the mid-2010s, the number of transcriptomics sequencing studies on mungbean increased sharply (Moe et al., 2011; Chen et al., 2015; Li et al., 2015; Lin et al., 2016; Liu C. et al., 2016; Liu M. S. et al., 2016; Tian et al., 2016; Kumar et al., 2020; Chang et al., 2021; Dasgupta et al., 2021; Hu et al., 2022; Sreeratree et al., 2022; Sudha et al., 2022; Zhao et al., 2022). The majority of these transcriptome analyses were related to responses to biotic and abiotic stresses, for example, drought, chilling temperature, waterlogging, heat, bruchids (*C. chinensis*), and Fusarium wilt (*Fusarium oxysporum*).

## QTL Mapping and (Candidate) Gene Discovery in Mungbean

Quantitative trait loci mapping and gene cloning play pivotal roles in understanding the genetic basis of variation in quantitative and complex traits that are important in molecular breeding. QTLs can be statistically localized onto genetic linkage maps by exploiting linkage relationships between markers and phenotypes in experimental populations. Several traits in mungbean have been dissected by QTL mapping (Table 1). In this review, we focus on some important traits, as follows.

**Table 1:** Quantitative trait loci identified for various traits in mungbean by gene mapping using experimental populations.

**Trait**	**Population(s)**	**DNA markers**	**Number of QTLs and name(s) (if available)**	**References**
Powdery mildew	F_2_ (TC1966 × VC3980A)	RFLP	3	(Young et al., 1993)
	F_2_ (TC1966 × VC1210A)	RFLP, AFLP	2; *PMR1, PMR2*	(Chaitieng et al., 2002)
	RIL (Berken × ATF3640)	RFLP	1	(Humphry et al., 2003)
	RIL (KPS1 × VC6468-11-1A)	SSR	2; *qPMR-1, qPMR-2*	(Kasettranan et al., 2010)
	F_2_ (KPS1 × V4718)	SSR	3; *qPMV4718-1, qPMV4718-2, qPMV4718-3*	(Chankaew et al., 2013)
	F_2_ and BC_1_F_1_ (CN60 × RUM5)	SSR	3; *qPMRUM5-1, qPMRUM5-2, qPMRUM5-3*	(Chankaew et al., 2013)
	RIL (CN72 × V4718)	ISSR, ISSR-RGA	1; *qPMC72V18-1*	(Poolsawat et al., 2017)
Cercospora leaf spot	F_2_ (KPS1 × V4718)	SSR	1; *qCLS*	(Chankaew et al., 2011)
	RIL (CN72 × V4718)	SSR, ISSR, ISSR-RGA	1; *qCLSC72V18-1*	(Arsakit, 2019)
Yellow mosaic disease	RIL (NM92 × TC1966)	RAPD, AFLP, SCAR, CAP, SSR	4; *MYMIVr 7_104, MYMIVr 8_48.8, MYMIVr 9_6.4, MYMIVr 9_25*	(Chen et al., 2013)
	RIL (KPS2 × NM10-12-1)	AFLP, SSR	5; *qMYMIV1, qMYMIV2, qMYMIV3, qMYMIV4, qMYMIV5*	(Kitsanachandee et al., 2013)
	F_2_ and BC_1_F_1_ (BM1 × BM6)	RGA, SCAR, SSR	2; *qMYMIV2, qMYMIV7*	(Alam et al., 2014b)
	RIL [VRM (Gg) 1 × TNAU RED]	SNP	4; *qMYMV4_1, qMYMV5_1, qMYMV6_1*; *qMYMV10_1*	(Mathivathana et al., 2019)
Bruchid	F_2_ (VC3890 × TC1966)	RFLP	1; *Br*	(Young et al., 1992)
	BC_20_F_2_ (Osaka-ryokuto × TC1966)	RFLP, RAPD	1; *Br*	(Kaga and Ishimoto, 1998)
	RIL (Berken × ACC41)	RFLP	1; *Br2*	(Mei et al., 2009)
	RIL (NM92 × TC1966)	RAPD, AFLP, SCAR, CAP, SSR	3; *B^*r*^ 7_114, B^*r*^ 7_132, B^*r*^ 9_26*	(Chen et al., 2013)
	F_2_ (Sunhwa × Jangan)	RAPD, CAP, SSR, STS	2	(Hong et al., 2015)
	RIL (Berken × ACC41)	RFLP, SSR, STS	1; *Br1*	(Wang et al., 2016)
	BC_11_F_2_ (KPS1 × V2802)	SSR, STS	1; *qBr*	(Chotechung et al., 2016)
	RIL (TC1966 × NM92)	SNP, SSR, CAP	1	(Schafleitner et al., 2016)
	RIL (V2802 × NM94)	SNP, SSR, CAP	2	(Schafleitner et al., 2016)
	BC_11_F_2_ (KPS1 × V2709)	SSR, InDel	1; *qBr5.1*	(Kaewwongwal et al., 2017)
	F_2_ (Jilyu7 × V1128)	SSR, STS, InDel	1; *qBr3.1*	(Liu et al., 2018)
	F_2_ (KPS2 × ACC41)	SSR, STS, InDel	2; *qBr5.1, qBr5.2*	(Kaewwongwal et al., 2020)
Bean bug	BC_20_F_2_ (Osaka-ryokuto × TC1966)	RFLP, RAPD	1; *Br*	(Kaga and Ishimoto, 1998)
	F_2_ (Sunhwa × Jangan)	RAPD, CAP, SSR, STS	1	(Hong et al., 2015)
Seed weight	F_2_ (TC1966 × VC3980A)	RFLP	4	(Fatokun et al., 1992)
	RIL (Berken × ACC41)	RFLP	11, *swA, swB1, swB2, swD, swE1, swE2, swF, swG, swI, swJ, swK*	(Humphry et al., 2005; Mei et al., 2009)
	BC_1_F_1_ (JP229096 × JP211874)	SSR	7; *Sd100wt5.1.1+, Sd100wt5.1.2+, Sd100wt5.2.1+, Sd100wt5.3.1+, Sd100wt5.7.1+, Sd100wt5*.8.1+, *Sd100wt5.11.1+*	(Isemura et al., 2012)
	F_2_ (KUML29-1-3 × W021)	SSR	6; *Sd100wt2.1, Sd100wt2.2, Sd100wt4.1, Sd100wt8.1, Sd100w9.1, Sd100wt11.1*	(Kajonphol et al., 2012)
	F_2_ (V1725BG × AusTRCF321925)	SSR	5; *SD100WT1.1, SD100WT2.1, SD100WT8.1, SD100WT9.1, SD100WT10.1*	(Sompong et al., 2012)
	RIL (NM92 × TC1966)	RAPD, AFLP, SCAR, CAP, SSR	3; *HSW 1_78, HSW 3_16, HSW 9_50*	(Chen et al., 2013)
	F_2_ and BC_1_F_1_ (BM1 × BM6)	SSR	4; *qSDW1.1, qSDW6.1, qSDW8.1, qSDW9.1,*	(Alam et al., 2014a)
	F_2_ (KPS1 × V4718)	SSR	6; *qSW2.1, qSW2.2, qSW4.1, qSW5.1, qSW10.1, qSW11.1*	(Somta et al., 2015)
Flowering time	BC_1_F_1_ (JP229096 × JP211874)	SSR	4; *Fld5.2.1–, Fld5.4.1–, Fld5.6.1–, Fld5.11.1+*	(Isemura et al., 2012)
	F_2_ (KUML29-1-3 × W021)	SSR	4; *Fld2, Fld4.1, Fld4.2, Fld11*	(Kajonphol et al., 2012)
	F_2_ (V1725BG × AusTRCF321925)	SSR	3; *DFL4.1, DFL4.2, DFL7.1*	(Sompong et al., 2012)
	F_2_ (KPS1 × V4718)	SSR	5; *qDFL2.1, qDFL2.2, qDFL4.1, qDFL5.1, qDFL6.1*	(Somta et al., 2015)
	RIL (VC2917 × ZL)	SSR	8; *qFLD4.1, qFLD4.2, qFLD4.3, qFLD4.4, qFLD4.5, qFLD4.6, qFLD4.7, qFLD4.8*	(Liu et al., 2017)
	RIL (VC1973AG × V2984)	SNP	2; *Dff3*-*1* (*Df3*-*1, FI4-1*), *FI4-1*	(Hwang et al., 2017; Ha et al., 2021)
Maturity	BC_1_F_1_ (JP229096 × JP211874)	SSR	6; *Pddm5.2.1-, Pddm5.4.1-, Pddm5.6.1-, Pddm5.7.1+, Pddm5.9.1-, Pddm5.11.1+*	(Isemura et al., 2012)
	F_2_ (KUML29-1-3 × W021)	SSR	3; *Pddm2, Pddm4.1, Pddm4.2*	(Kajonphol et al., 2012)
	F_2_ (V1725BG × AusTRCF321925)	SSR	3; *DMT4.1, DMT7.1*	(Sompong et al., 2012)
	RIL (VC1973AG × V2984)	SNP	2; *SPM4-1, SPM7-1*	(Ha et al., 2021)
Plant height	BC_1_F_1_ (JP229096 × JP211874)	SSR	6; *Stl5.1.1+, Stl5.2.1+, Stl5.3.1+, Stl5.6.1+, Stl5.9.1–, Stl5.10.1+*	(Isemura et al., 2012)
	RIL (KPS2 × NM10-12)	SSR, AFLP	4; *qPH1.1_combined, qPH2.1_combined, qPH3.1_combined, qPH4.1_combined*	(Somta et al., 2014)
	RIL (VC2917 × ZL)	SSR	16; *qPH2.1, qPH3.1, qPH4.1, qPH4.2, qPH4.3, qPH4.4, qPH4.5, qPH4.6, qPH4.7, qPH5.1, qPH5.2, qPH5.3, qPH5.4, qPH8.1, qPH8.2, qPH8.3*	(Liu et al., 2017)
	RIL (VC1973AG × V2984)	SNP	2; *Height4-1, Height5-1*	(Ha et al., 2021)
Seed yield	RIL (KPS2 × NM10-12)	SSR, AFLP	3; *qSY2.1_combined, qSY3.1_combined, qSY9.1_combined*	(Somta et al., 2014)
	RIL (VC2917 × ZL)	SSR	4; *qYLD1.1, qYLD4.1, qYLD8.1, qYLD8.2*	(Liu et al., 2017)
Drought tolerance	RIL (VC2917 × ZL)	SSR	2; *qPHI4.1, qPHI4.2*	(Liu et al., 2017)
Calcareous soil resistance/iron deficiency chlorosis	F_2_ (KPS1 × NM10-12)	AFLP	1; *IR*	(Srinives et al., 2010)
	RIL (KPS2 × NM10-12)	SSR, AFLP	2; *qIDC2.1, qIDC3.1*	(Prathet et al., 2012)
Seed dormancy	RIL (Berken × ACC41)	RFLP	4; *HsA, HsB, HsC, HsK*	(Humphry et al., 2005)
	BC_1_F_1_ (JP229096 × JP211874)	SSR	4; *Sdwa5.1.1+, Sdwa5.2.1+, Sdwa5.3.1+, Sdwa5.4.1+*	(Isemura et al., 2012)
	F_2_ (KPS2 × ACC41)	SSR, InDel	2; *Sdwa5.1.1+, Sdwa5.1.2+*	(Laosatit et al., 2022)
Pod shattering	BC_1_F_1_ (JP229096 × JP211874)	SSR	2; *Pdt5.1.1-, Pdt5.7.1-*	(Isemura et al., 2012)
Seed phytic acid content	F_2_ (V1725BG × AusTRCF321925)	SSR	2; *SDPAP4.1, SDPAP11.1*	(Sompong et al., 2012)
Seed starch content	F_2_ (V6087AG × V5020BY)	SSR	1; *qSSC8.1*	(Masari et al., 2017)

### Bruchid Resistance

Bruchids, or seed weevils, are stored-product insects that infest the starchy seeds of various crops. Azuki bean weevil (*C. chinensis* L.) and cowpea weevil (*Callosobruchus maculatus* Fab.) are the most important bruchids in the storage of mungbean and several other legume crops (Srinives et al., 2007). Seed resistance to bruchids in mungbean is due to antibiosis in cotyledons (Somta C. et al., 2008) and is controlled by a single dominant locus, *Br*, with modifiers (Kitamura et al., 1988; Somta et al., 2007). *Br* was the first gene to be molecularly located on the mungbean genetic map (Young et al., 1992). The first fine-mapping study in mungbean attempted to narrow down the *Br* genomic region in wild mungbean “TC1966” (Kaga and Ishimoto, 1998) using RFLPs and RAPD markers. Several years later, map-based cloning of the *Br* locus in mungbean cultivar “Jangannogdu,” deriving resistance from landrace cultivar “V2709,” identified *VrBURP1*, encoding a protein containing the BURP (BNM2, USP, RD22, and PG1b) domain, as the gene responsible for resistance (Jeong et al., 2015). By combining transcriptomic, proteomic, and gene expression analyses of the near-isogenic line VC6089A carrying the *Br* locus from TC1966 and its recurrent parent VC1973A, gene *g5551*, encoding aspartic proteinase, and *g34458* and *g39185*, each encoding a protein containing a BURP domain, were identified as candidate genes for resistance (Lin et al., 2016). *g34458* and *g39185* are located on mungbean chromosome 5 near the *Br* locus (Lin et al., 2016; Liu M. S. et al., 2016; Schafleitner et al., 2016). The protein sequences encoded by *VrBURP1, g34458*, and *g39185* are similar, and they all match polygalacturonase (Laosatit et al., 2020).

Chotechung et al. (2016) exploited information from fine mapping of the *Br* locus in TC1966 (Kaga and Ishimoto, 1998) and the draft genome sequence of mungbean to find candidate genes for resistance in the mungbean landrace “V2802,” and also found that the *Br* locus is localized on chromosome 5 between markers VrBr-SSR013 and DMB-SSR158. DMB-SSR158 co-segregated perfectly with resistance. VrBr-SSR013 and DMB-SSR158 corresponded to *Vradi05g03940* (*VrPGIP1*) and *Vradi05g03950* (*VrPGIP2*), respectively. No other genes were found between these two genes. *VrPGIP1* and *VrPGIP2* each encode a polygalacturonase inhibitor (polygalacturonase-inhibiting protein; PGIP). Sequence alignment of *VrPGIP1* and *VrPGIP2* between the mapped mungbean parents revealed SNPs in only the coding sequence of *VrPGIP2*. The SNPs cause amino acid changes in the VrPGIP2 protein in V2802. The same resistance allele is also found in other bruchid-resistant mungbean, including TC1966, “V1128,” “V2066,” and “V2817.” Later, new resistance alleles of *VrPGIP2* and/or *VrPGIP1* were identified in the cultivated mungbean accession “V2709” and wild mungbean accession “ACC41” (Kaewwongwal et al., 2017, 2020). Polygalacturonase is an important digestive enzyme secreted in the gut of *C. maculatus* (Nogueira et al., 2012). Recently, VrPGIP1 and VrPGIP2 proteins isolated from V2802 were shown to enzymatically inhibit polygalacturonases (PGs) isolated from larvae and adults of *C. maculatus* (Zhang et al., 2021). A feeding test also confirmed the antibiosis effect of the proteins VrPGIP1 and VrPGIP2 against bruchids (Zhang et al., 2021). These results strongly indicate that *VrPGIP1* and *VrPGIP2* are the genes at the *Br* locus that confer resistance to bruchids in seeds by inhibiting *C. maculatus* PGs to degrade pectin and the cell wall of seeds, thus limiting energy and other digestive enzymes to access their substrates in the ingested seeds. *VrPGIP1* and *VrPGIP2* represent a new class of insect resistance genes in plants.

### Powdery Mildew Resistance

*Erysiphales* fungi are obligate biotrophic plant pathogens that produce white mycelia on the leaves, stems, and fruits of angiosperm plants. Powdery mildew (PM) disease caused by obligate fungus *E. polygoni* is a common leaf disease of mungbean crops grown in different regions. The disease is also caused by *Sphaerotheca phaseoli* (Z. Y. Zhao) U. Braun (Lee et al., 2002), *Podosphaera xanthii* (Castagne) U. Braun and Shishkoff (Sheu et al., 2021), and *Erysiphe vignae* sp. nov. (Kelly et al., 2021). The disease is prevalent in cool and dry environments and is favored by high-density plant populations and cloudy weather (Laosatit et al., 2020). It can cause reduced seed yield of susceptible mungbean cultivars by up to 40% if there is no protection.

Young et al. (1993) was the first to identify three QTLs for partial resistance in “VC3890A” using RFLP markers. Nearly 10 years later, Chaitieng et al. (2002) used RFLP and amplified fragment length polymorphism (AFLP) markers and identified one major and one minor QTL, *PMR1*, and *PMR2*, controlling resistance to PM in “VC1210A.” *PMR1* explained up to 65% of the disease resistance variation. A major QTL governing resistance in “ATF3640” was mapped using RFLP markers (Humphry et al., 2003). This QTL accounted for up to 86% of the variation in disease reaction. SSR and sequence-tagged site (STS) markers were added to the QTL region identified in ATF3640 (Zhang et al., 2008). Using SSR markers, two major QTLs, *qPMR1* and *qPMR2*, were detected for PM resistance in “VC6468-11-1A” (Kasettranan et al., 2010). These two QTLs accounted for 20.10 and 57.81% of the variation in disease reaction, respectively. Chankaew et al. (2013) located QTLs controlling resistance in highly resistant accessions “RUM5” and “V4718.” Two major (*qPMRUM5-2* and *qPMRUM5-3*) and one minor (*qPMRUM5-1*) QTL were detected in RUM5, while one major (*qPMV4718-2*) and two minor (*qPMV4718-1* and *qPMV4718-3*) QTLs were identified in V4718. Comparative linkage analysis of the QTLs for PM resistance in different mungbean accessions revealed that *qPMRUM5-3* in RUM5, *qPMV4718-3* in V4718, *qPMR2* in VC6468-11-1A, and the resistance QTL in ATF3640 are in the same linkage group and possibly the same locus, whereas *qPMRUM5-2* in RUM5 and *qPMR1* in VC6468-11-1A are on the same linkage group and very likely the same locus (Chankaew et al., 2013). In contrast to the above studies involving *E. polygoni*, Arsakit (2019) showed that the resistance to PM caused by *S. phaseoli* in V4718 is controlled by a single major QTL, *qPMC72V18*, localized on linkage group 3 between SSR markers VR108 and VR393. *qPMC72V18* was on a different linkage group from other QTLs for PM resistance.

*Mildew locus O* (*MLO*) genes are known to be involved in susceptibility/resistance to PM diseases in plants. In fact, *MLO*s are susceptibility genes (*S*-genes), genes that facilitate infection and support compatibility for a pathogen (van Schie and Takken, 2014). Loss of *MLO* function is associated with resistance to the disease and is known as *mlo*-based resistance (Kusch and Panstruga, 2017). In barley, *mlo*-based resistance has been durable under field conditions and has been utilized for over 40 years with no reported breakdown of resistance (Kusch and Panstruga, 2017). The binding of Ca^+^-dependent calmodulin (CaM) to the CaMBD domain of the MLO protein promotes susceptibility to PM during infection (Consonni et al., 2006). Mutation in the CaMBD domain, which reduces or abolishes CMD binding, results in resistance to disease. Eighteen *MLO* genes have been identified in the mungbean reference genome (Rispail and Rubiales, 2016). Bioinformatic analysis and fine mapping of *qPMRUM5-3*, which confers PM resistance in RUM5, demonstrated that *VrMLO12* is the only candidate gene at the *qPMRUM5-3* (Yundaeng et al., 2020). Some SNPs in the *VrMLO12* of RUM5 cause amino acid changes in functional domains, transmembrane 6 (TM6) domain, and calmodulin-binding domain (CaMBD) of the VrMLO12 protein. Similar mutations have also been found in V4718. Therefore, amino acid change in the CaMBD domain of VrMLO12 is likely the cause of PM resistance in RUM5 and V4718, although the amino acid change in TM6 may also contribute to the resistance. The *mlo*-based resistance appears to be a major PM resistance mechanism in mungbean.

Mildew locus O proteins from plants and algae are classified into seven clades, I to VII (Pessina et al., 2016). Interestingly, in contrast to the finding in other plant species that all MLO proteins involved in susceptibility/resistance to PM disease are restricted to clade IV (for monocots) and clade V (for dicots), VrMLO12 belongs to MLO clade II (Rispail and Rubiales, 2016; Yundaeng et al., 2020). It has been proposed that MLO proteins negatively regulate vesicle-associated and actin-dependent defense pathways at the site of powdery mildew fungi penetration (Panstruga, 2005). The secretory vesicle traffic modulates pathogen penetration, leading to the formation of papillae or cell wall appositions (Feechan et al., 2011), which are associated with *mlo*-based resistance (Consonni et al., 2006). It is not yet known how VrMLO12 modulates susceptibility to *E. polygoni*. Molecular characterization of the function of VrMLO12 could allow a better understanding of the interaction between MLO proteins and powdery mildew fungi, and thus of *mlo*-based resistance.

Resistance to PM appears to be controlled by several loci. To date, however, only one gene, *VrMLO12*, has been identified as a candidate gene for resistance. Additional attempts should be made to identify candidate genes for other loci for resistance, for example, the genes for *qPMRUM5-2* that express stable resistance. Since the loss-of-function mutations in clade V MLO genes lead to broad-spectrum resistance to PM in several plant species, the clade V *MLO* genes in mungbean, namely *VrMLO1, VrMLO2, VrMLO5, VrMLO6, VrMLO11*, and *VrMLO17* (Rispail and Rubiales, 2016), can be suitably targeted as selected candidate genes for association analysis of resistance to PM disease in mungbean. They could also be candidates for the development of *mlo*-based resistance in mungbean through genetic engineering or gene editing.

### Cercospora Leaf Spot Resistance

Cercospora leaf spot (CLS) disease is an important foliar disease in mungbean crops grown in tropical and subtropical regions. The disease is caused by the obligate biotrophic fungus *Cercospora canescens* Ellis and Martin and occurs widely in wet seasons (Laosatit et al., 2020). This fungus causes defoliation and up to 50% seed yield reduction in mungbean. Despite its importance, there have been only a few studies reporting the genetics and genomics of resistance to CLS disease in mungbean. All of those studies used the same resistant germplasm, V4718, which is also resistant to PM. CLS resistance in V4718 is controlled by a single resistance gene (Chankaew et al., 2011). A single and stable major QTL, *qCLS*, controlling resistance was mapped onto linkage group 3 between SSR markers CEDG117 and VR393 (Chankaew et al., 2011). The location of *qCLS* was validated in an independent study (Arsakit, 2019). Fine mapping targeting the CEDG117-VR393 region in an F_2_ population and an advanced back-cross population was carried out, and the *qCLS* was consistently narrowed down to a genome region of 13.0 kb on chromosome 6 containing only a single gene, *LOC106765332* (*VrTAF5*), which encodes general transcription initiation factor IID (TFIID) subunit 5 [TATA-box-binding protein (TBP)-associated factor 5 (TAF5)] (Yundaeng et al., 2021). A *VrTAF5* sequence comparison between V4178 and KPS1 revealed an SNP in the coding sequence. This SNP causes an amino acid change in VrTAF5. These results indicate that *VrTAF5* is the gene at the *qCLS* locus and is responsible for resistance to *C. canescens*. This is the first line of evidence showing an association between TAF5 and stress resistance in plants. Since TAF5 is a key protein in the formation of the TFIID and Spt-Ada-Gcn5 acetyltransferase (SAGA) complexes, which regulate gene expression (Soffers and Workman, 2020), VrTAF5 possibly modulates the expression of resistance genes to *C. canescens*. It is worth noting that *qCLS* is co-localized with *qPMC72V18*, conferring PM resistance caused by *S. phaseoli* in V4718 (Arsakit, 2019). It is tempting to speculate that *VrTAF5* controls resistance to both *C. canescens* and *S. phaseoli*.

### Yellow Mosaic Disease

Yellow mosaic disease (YMD) is the most important and devastating disease in mungbean production in South Asia and is becoming a major threat in Myanmar and Thailand. YMD is caused by three species of begomovirus: mungbean yellow mosaic virus (MYMV), mungbean yellow mosaic India virus (MYMIV), and horse gram yellow mosaic virus (HgYMV). These viruses are transmitted and spread *via* whitefly (*Bemisia tabaci* Gennadius). The symptoms of YMD include leaf yellowing and/or chlorosis followed by leaf necrosis, fewer pods, small and abnormal pods and seeds, and stunted plants. YMD causes up to 100% yield loss. Resistance to YMD in mungbean has been reported to be controlled by a single dominant gene or a single recessive gene, or two recessive genes or complementary recessive genes (reviewed in Laosatit et al., 2020).

Quantitative trait loci mapping in an RIL population in India revealed four QTLs for resistance, with QTL *MYMIVr9_25* showing the largest effect, and only QTL *MYMIVr8_48.8* was consistently identified across different years (Chen et al., 2013). Evaluating MYMIV resistance of an RIL population in India and Pakistan, Kitsanachandee et al. (2013) detected three QTLs, *qMYIMV1, qMYIMV2*, and *qMYIMV3*, for resistance in India and two QTLs, *qMYIMV4* and *qMYIMV5*, in Pakistan. All of the QTLs except *qMYMIV4* accounted for <20% of disease variation. Two major QTLs, *qMYMIV2.1* and *qMYMIV7.1*, for MYMIV resistance in two locations in Bangladesh were consistently identified using F_2_ and BC_1_F_1_ populations (Alam et al., 2014a). Each QTL accounted for more than 40% of disease resistance variation.

When the reference mungbean genome sequence became available, Mathivathana et al. (2019) used an RIL population of an interspecific cross between mungbean (VRM (Gg) 1, susceptible) and rice bean (TNAU RED, resistant) to map the QTLs for MYMV resistance using SNP markers. Two and three QTLs on chromosomes 4, 5, 6, and 10 were identified for the years 2015 and 2016, respectively. The QTL *qMYMV4_1* on chromosome 4 was identified in both years and showed phenotypic variance of about 20%. The physical distance of markers VigSNP_04_32 and VigSNP_04_36 flanking *qMYMV4_1* was about 1.28 Mb and contained 83 annotated genes. Laosatit et al. (2020) explored the linkages of QTLs for YMD resistance and found that the QTLs *MYMIVr9_25, qMYMIV4* (*qMYMIV1*), and *qMYMIV2.1* were mapped to the same linkage group and were possibly the same QTL because the resistance sources used in all studies derived their resistance from the same germplasm. If this is the case, this QTL will be useful for breeding YMD-resistant mungbean cultivars, since it is stable across environments. One of the QTLs derived from rice bean, *qMYMV5_1*, was located in the *qMYMIV2.1* region (Mathivathana et al., 2019). This suggests a common mechanism of resistance to YMD between mungbean and rice bean.

A transcriptome analysis was conducted to identify genes involved in MYMV resistance in the resistant mungbean “VGGRU-1,” which is a derivative of the cross VRM (Gg) 1 × TNAU RED (Sudha et al., 2022), by comparing the differentially expressed genes (DEGs) between VGGRU-1 and VRM (Gg) 1. We checked those DEGs and found that *Vradi04g06840* is of interest, as it encodes a protein similar to suppressors of TYPE ONE PROTEIN PHOSPHATASE 4 MUTATION (TOPP4-1) (SUT). SUT1 is a nucleotide-binding, leucine-rich repeat protein of the C-NB-LRR type and is involved in TOPP4-mediated immune response in Arabidopsis (Yan et al., 2019). In addition, *Vradi04g06840* is within the *qMYMV4_1* region, and therefore can be considered a lead candidate for further molecular investigation of resistance.

### Seed Size (Seed Weight)

Seed size directly contributes to seed yield and is important in the mungbean industry. Farmers, processors, and buyers prefer large-seeded mungbean. The trait has certainly been a major target for selection by farmers and breeders. Seed weight is a polygenic but highly heritable trait with an *H*^2^ value of more than 80% (Humphry et al., 2005; Yimram et al., 2009; Isemura et al., 2012). Together with PM and CLS, seed weight was one of the first traits molecularly mapped in mungbean (Fatokun et al., 1992). There have been some studies on QTLs controlling seed weight, most of them using populations derived from hybridization between wild and cultivated lines, and 4–11 QTLs were identified for the trait (Fatokun et al., 1992; Humphry et al., 2005; Mei et al., 2009; Isemura et al., 2012; Kajonphol et al., 2012; Sompong et al., 2012; Alam et al., 2014b; Somta et al., 2015).

In the study of Alam et al. (2014b), four QTLs, *qSDWT1.1, qSDWT6.1, qSDWT8.1*, and *qSDWT9.1*, were consistently identified for seed weight in an F_2:3_ population grown in three environments. They noted that (i) *qSDWT1.1* and *qSDWT8.1* appeared to be the same as QTLs *Sd100w5.1.1* and *Sd100w8.1.1* reported by Isemura et al. (2012) and the same as the QTLs on LG VI and LG II, identified by Fatokun et al. (1992); (ii) *qSDWT8.1* and *qSDWT9.1* corresponded to the QTLs *Sd100wt8* and *Sd100wt9*, identified by Kajonphol et al. (2012); and (iii) *qSDWT1.1, qSDWT8.1*, and *qSDWT9.1* corresponded to the QTLs *SD100WT1.1, SD100WT8.1*, and *qSDWT9.1* reported by Sompong et al. (2012). Some mungbean seed weight QTLs are conserved in other related legume species, such as azuki bean, rice bean, and cowpea (Fatokun et al., 1992; Isemura et al., 2012; Alam et al., 2014b; Somta et al., 2015). For instance, *qSDWT1.1* was also found in all of these legume crops, *qSDWT6.1* was identified in azuki bean and cowpea, *qSDWT8.1* was found in cowpea, and *qSDWT9.1* was detected in azuki bean (Alam et al., 2014b). Since *qSDWT8.1* expressed the largest or second-largest effect (Isemura et al., 2012; Sompong et al., 2012; Alam et al., 2014b), this QTL should be further studied to identify genes underlying seed size.

### Plant Architecture and Phenology

Stem determinacy (determinate growth) is an agronomically important trait that is selected during mungbean domestication and breeding, as it has been useful for farmers since ancient times when harvesting mungbean pods both by hand and using machines (Isemura et al., 2012). Stem determinacy, flowering time, and photoperiod insensitivity have been genetically improved by breeders to develop modern mungbean cultivars with a short flowering period and early maturity for the ease of harvest (Fernandez and Shanmugasundaram, 1988). In general, improved mungbean cultivars have a determinate habit, whereas all wild mungbean germplasms have an indeterminate habit. The determinate habit is recessive and is controlled by a single gene, originally named *stdet5.9.1*, and mapped onto linkage group 9 between SSR markers GATS11 and CEDG172 (Isemura et al., 2012). Later, *stdet5.9.1* was renamed *VrDet1* (Li et al., 2018). In soybean, stem determinacy is controlled by *Dt1*, which is orthologous to Arabidopsis *Terminal Flowering 1* (*TFL1*), encoding a shoot apical meristem signaling protein (Liu et al., 2010). *Vradi03g04510* (*VrDet1*) in mungbean is a *TFL1* ortholog (Li et al., 2018). Sequencing of the *VrDet1* gene and its upstream region between cultivated mungbean “Dahyeon” and wild mungbean “W164” revealed no polymorphism in exons, but 15 SNPs, 12 of which are in the promoter region. Association analysis revealed perfect co-segregation between *VrDet1* and the stem determinacy trait. Compared to wild (indeterminate growth) mungbean, *VrDet1* is much less expressed in cultivated (determinate growth) mungbean. Complementation tests showed that the *VrDet1* allele from cultivated mungbean complements *TFL1* function in Arabidopsis, whereas the *VrDet1* allele from wild mungbean does not, and that mutations in the promoter region of *VrDet1* causes the change from indeterminacy in wild mungbean to determinacy in cultivated mungbean. A *VrDet1* and upstream sequence comparison between 13 wild and 14 cultivated mungbean accessions revealed three unique SNPs in the promoter region between the two groups. Further investigation elucidated that only an SNP belonging to a cis-regulatory element of MARTBOX and one belonging to a NODCON2GM/OSE2ROOTNODULE cis-regulatory element were essential for *VrDet1* function and indeterminacy. These results indicate that the transition from indeterminacy to determinacy in cultivated mungbean is due to the loss-of-function mutations in the two cis-regulatory elements in the promoter region of *VrDet1*. It is believed that the SNPs in the cis-regulatory elements in cultivated mungbean reduce or cancel the expression of *VrDet1*, thereby suppressing the expression of floral identity genes in the apical meristems of stem tips in determinate mungbean to produce terminal flowers that develop into terminal pods (Li et al., 2018).

Photoperiod and temperature are key environmental factors that affect the phenological development of mungbean in all growth stages, and thus determine yield and adaptability. Mungbean is a quantitative short-day (SD) and warm-season plant that can be cropped in a wide range of latitudes between 51°N and 40°S. Many landraces and most wild mungbean are sensitive to photoperiod, whereas modern cultivars are less sensitive to photoperiod and are early flowering. Flowering time is a quantitative trait and is highly correlated with maturity time. Understanding the genetic mechanism underlying the photoperiod response in mungbean is crucial for expanding mungbean production to high-latitude regions.

Four QTLs on LGs 2, 4, and 11 were identified for days to first flowering (DFL) in an F_2_ population of a cross between cultivated mungbean showing early flowering (DFL = 31 days) and wild mungbean showing late flowering (DFL = 66 days) grown during SD and long-day (LD) conditions in Thailand (Kajonphol et al., 2012). *Fld2* on LG2 and *Fld4.2* on LG4 were the major QTLs, contributing 15.9 and 28.6% of the flowering variation. Similarly, four QTLs each on LGs 2, 4, 6, and 11 were detected for DFL in an F_2_ population of a cross of wild and late-flowering mungbean (DFL = 81 days) × cultivated and early-flowering mungbean (DFL = 35 days) grown from July (day length about 14 h) to December (day length about 10 h) 2007 (Isemura et al., 2012). The *Fld5.2.1-* on LG2 had the largest effect (PVE = 32.9%), followed by that on LG4 (PVE = 24.0%). A study of an F_2_ population of a cross between cultivated mungbean (DFL = 34 days) and wild mungbean (DFL = 44 days) grown in summer (LD condition) in Thailand revealed three QTLs on LGs 4 and 7 governing DFL (Sompong et al., 2012). *DFL4.1* and *DFL4.2* on LG4 were the major loci, accounting for 33.4 and 25.7% of the flowering variation, respectively. In another study, using an F_2_ population of a cross between early- and late-flowering cultivated mungbean evaluated in three seasons (one LD and two SD conditions) in Thailand, five QTLs on LGs 2, 4, 5, and 6 were identified for rainy seasons and two QTLs on LG2 were detected for dry seasons (Somta et al., 2015). These QTLs accounted for between 12.6% (*qDFL2.2*) and 32.0% (*qDFL5.1*) of the flowering variation. It should be noted that there was a 11-day difference between the parents in DFL under LD conditions, but they flowered on the exact same or nearly the same day under SD conditions. The QTLs on LGs 2, 4, 6, and 11 were always detected, especially those on LGs 2 and 4. Two QTLs on LG2 were always detected in both SD and LD conditions, indicating their importance in regulating flowering in mungbean, while the QTLs on LGs 4, 6, and 11 were responsive to LD.

A BLASTN search for chromosomal locations of SSR markers surrounding the QTLs on LG2 against the genome sequence of wild mungbean “NI1135” (https://viggs.dna.affrc.go.jp) revealed a physical association between the markers on LG2 and a *Phytochrome* gene, and between the markers on LG4 and *Flowering Locus T*-like (*FT-*like), *Flowering Locus K homology domain* (*FLK*), *APETALA2*/*Ethylene-Responsive Factor* (*AP2*/*ERF*), and *CONSTANS* genes (Somta, unpublished data). *FT* encodes florigen, which serves as an integrator of flowering time control in several plant species. In soybean, FT-like genes *GmFT2a* and *GmFT5a* promote flowering, whereas *GmFT1a* and *GmFT4* inhibit flowering and maturity (reviewed in Lin et al., 2021). In Arabidopsis, *FLK* positively regulates flowering by repressing the expression of a flowering-time gene (Mockler et al., 2004). AP2/ERF proteins are key regulators of various growth and development processes, including reproductive and vegetative organ development (Xie et al., 2019), whereas CONSTANS plays a central role in integrating various external and internal signals into a photoperiodic flowering pathway (Shim et al., 2017). Correlative relationships between these genes and flowering time in mungbean should be molecularly confirmed.

In addition to the low-resolution mapping of QTLs for DFL mentioned above, high-resolution QTL mapping for DFL was carried out using 1,321 SNPs in an RIL population derived from a cross between the elite breeding line VC1973A (DFL = 38 days) and the landrace mungbean V2984 (DFL = 44 days) grown under LD conditions in Korea (Hwang et al., 2017). Only a single QTL, *Dff 3*-*1*, was mapped on chromosome 3. *Dff 3*-*1* accounted for 33.4% of the FLD variation detected, and *Vradi03g07170* (*VrPHYA*), encoding phytochrome A (PHYA), was identified as a candidate gene for this locus (Hwang et al., 2017). *VrPHYA* is homologous to soybean *E3* (*GmPHYA3*) and *E4* (*GmPHYA2*) genes, known to be involved in the control of flowering under LD conditions (reviewed in Lin et al., 2021). In a recent study, high-resolution mapping of QTLs for plant height (PLH), flower initiation (FLI), number of branches (BRN), number of nodes (NDN), and synchronous pod maturity (SPDM) was carried out using 8,966 SNPs in an RIL population grown in Korea (Ha et al., 2021). This RIL population was possibly the same one used by Hwang et al. (2017). QTL analysis detected one major QTL on chromosome 4 and one minor QTL on another chromosome for all the traits except BRN, for which only one major QTL on chromosome 3 was detected. The QTLs on chromosome 4 detected for PLH, FLI, NDN, and SPDM were in the same or nearly the same region, suggesting a pleiotropic effect on these traits. Based on the newly assembled genome of VC1973A, candidate genes for the major QTLs of these traits were identified. *Vradi04g00002773* (*VrPHYA* in Hwang et al., 2017) was identified as the candidate gene for PLH, BRN, and SPDM, while *Vradi04g00002764* (*VrBONSAI*), encoding a protein with similarity to the APC13 component of the anaphase-promoting complex, and *Vradi03g00002294* (*VrERF4*), producing ERF4, were identified as candidate genes for FLI and BRN, respectively.

In *Arabidopsis*, as compared to wild type, *bonsai* plants are shorter and with more compact inflorescence, resulting in reduced PLH (Saze and Kakutani, 2007). In *Arabidopsis*, ERF4 is a negative regulator that can modulate ethylene and abscisic acid responses. The *ERF4* gene appears to affect plant senescence. Transgenic *Arabidopsis* lines overexpressing *ERF4-R* showed slightly delayed flowering and silique emergence (Riester et al., 2019). Candidate genes *Vradi09g00002773* (*VrPHYA* homolog) and *Vradi11g00000623* (*VrELF3*), *Vradi05g00000394* (*VrPKpl*), and *Vradi07g00000916* (*VrHAK1*) were identified for minor QTLs controlling for PLH, NDN, and SPDM, PLH, and SPDM (Ha et al., 2021). All of these candidate genes are likely to have experienced breeding selection in the process of mungbean breeding that transformed landraces into modern cultivars with early flowering and maturity, compact plant habit, and synchronous pod maturity. Nonetheless, although *VrPHYA* appears to be useful in increasing early flowering (FLI and DFL) and SPDM, it decreases PLH and NDN. The pleiotropy of *VrPHYA* must be carefully considered to allow its exploitation in mungbean breeding. The genomes of mungbean and soybean are highly conserved (Kang et al., 2014; Yan et al., 2020). Soybean is molecularly well-studied for the photoperiodic regulation of flowering (Lin et al., 2021). The molecular mechanisms controlling photoperiod response and flowering in soybean can be used as a model in mungbean through comparative genomics. Mungbean genes corresponding to some key genes for the photoperiod response and flowering in soybean have been identified (Ha et al., 2021).

Quantitative trait loci mapping and candidate genes have also been reported for other traits related to plant architecture and phenology, including the morphology of leaves (Jiao et al., 2016; Wang et al., 2020), inflorescence (Lee et al., 2021), and flowers (Chen et al., 2016; Lin et al., 2020). Interestingly, candidate genes *Vradi04g00002481* for compound raceme, *Vradi04g00002442* (*Vradi03g04510* in Li et al., 2018) for stem determinacy, *Vradi04g00002773* (*Vradi03g07170* in Hwang et al., 2017) for DFL, PLH, BRN, and SPDM, and *Vradi04g00002764* for FLI are clustered on chromosome four. It would be difficult to break the close/tight linkage of these genes by gene recombination.

### Seed Dormancy

Non-dormancy of seeds and non-shattering of pods are the first two traits that are selected for the process of legume domestication. Nondormant seeds provide uniform germination and timely cultivation. In contrast, seed dormancy prevents germination of wild species in unsuitable environments. In legume and cereal crops grown in tropical and subtropical regions, the non-dormancy of seeds can result in preharvest sprouting, leading to yield and quality losses. Like other wild legume species, wild mungbean shows strong seed dormancy, which can provide protection against preharvest sprouting (Imrie et al., 1988; Lawn et al., 1988). Classical genetic studies suggested that seed dormancy in wild mungbean is controlled by a single dominant gene with or without modifying genes (Singh et al., 1983; Lawn et al., 1988). QTL analysis revealed that dormancy in wild mungbean ACC41 is controlled by one major and three minor QTLs, and only the major QTL, *HsA*, explaining 23.2% of the dormancy variation, could be confirmed for the trait (Humphry et al., 2005). *HsA* was located between RFLP markers cgO103 and VrCS364. Isemura et al. (2012) identified QTLs for domestication-related traits and found that seed dormancy in wild mungbean accession “JP211874” was conditioned by two major and two minor QTLs. *Sdwa5.1.1*+ showed the largest effect, explaining 33.7% of the seed dormancy variation. It was located between SSR markers cp05137 and CEDG074b. Laosatit et al. (2022) speculated that *HsA* and *Sdwa5.1.1*+ are the same loci and finely mapped *Sdwa5.1.1*+ using ACC41 as the source of seed dormancy; they found that the cp05137–CEDG074b interval contained two QTLs for dormancy, *Sdwa5.1.1*+ and *Sdwa5.1.2*+, with more or less equal effects. *Sdwa5.1.1*+ was located between markers VrSdp-SSR5 and VrKNAT7-SSR4, where there is only one gene, *Vradi07g13210* (*VrKNAT7-1*), in this genomic interval, while *Sdwa5.1.2*+ was localized between markers VrSdp-SSR102 and VrSdp-SSR104, which encompasses five genes: *Vradi07g10460, Vradi07g10470, Vradi07g10480, Vradi07g10490*, and *Vradi07g10500*.

Sequence alignment of *VrKNAT7-1* between ACC41 and KPS2 (cultivated mapping parent) revealed SNP and indel polymorphisms in CDS, 5′UTR and 3′UTR, and upstream sequences, although the SNPs in CDS cause synonymous mutation. Expression analysis of seed coat demonstrated higher expression of *VrKNAT7-1* and *Vradi06g06960* (*VrCYP86A*, a downstream gene of *VrKNAT7-1*) in ACC41 than in KPS2. *Vradi07g13210* encodes KNOTTED ARABIDOPSIS THALIANA 7 (KNAT7), a homolog of MtKNOX4 protein that controls physical seed dormancy by regulating the expression of *MtCYP86A*, which is involved in the cuticle biosynthesis pathway, and *MtKCS12*, which controls the production of very-long-chain lipids in the seed coat (Chai et al., 2016, 2021). Scarified seeds of ACC41 germinated rapidly under wet conditions, while the intact seeds of ACC41 remained dormant for several days. Microscopic observation revealed that ACC41 seeds possess a cuticle layer, while KPS2 seeds lack such a layer. These results indicate that *VrKNAT7-1* controls physical dormancy in wild mungbean.

At the *Sdwa5.1.2*+ region, containing five genes, *Vradi07g10480*, encoding calmodulin-like (CML) protein 1, is likely the gene controlling seed dormancy (Laosatit et al., 2022). Calmodulin (CaM) and CML proteins have been shown to be involved in the ABA-induced inhibition of seed germination and seedling growth. *Sdwa5.3.1*+, with PVE of about 14.2%, is another major QTL controlling seed dormancy in wild mungbean JP211874 (Isemura et al., 2012). This QTL is located between SSR markers GMES6583 and GMES0294a on LG3. We inspected the physical locations of these markers by blasting their primer sequences against the VC1973A genome and reanalyzed LG3 and *Sdwa5.3.1*+ by removing a few markers that seemed to be problematic and found that *Sdwa5.3.1*+ was mapped to nearly the same position as marker GMES0591 (Supplementary Figure 1). GMES0591 corresponds with the *LOC106764165* gene, encoding gibberellin 2-beta-dioxygenase 1 (GA2OX1), which controls gibberellin catabolism. The balance between GA and ABA biosynthesis and catabolism determines seed dormancy and germination (Finch-Savage and Leubner-Metzger, 2006). Therefore, both physical and physiological dormancies appear to control seed dormancy in wild mungbean.

Quantitative trait loci for bean bug (*Riptortus clavatus* Thunberg) resistance (Kaga and Ishimoto, 1998; Hong et al., 2015), seed phytic content (Sompong et al., 2012), iron-deficiency chlorosis (calcareous soil resistance) (Srinives et al., 2010; Prathet et al., 2012), drought tolerance (Liu et al., 2017), and domestication-related traits (Isemura et al., 2012; Kajonphol et al., 2012) have also been identified for mungbean. However, most of these QTLs underwent low-density mapping and have not been confirmed/validated.

## Genome-Wide Association Study

This study, also known as linkage disequilibrium (LD) mapping, is a form of gene mapping used to unravel the genetic basis of complex traits by identifying DNA markers, generally SNPs, associated with a particular trait of interest. GWAS takes advantage of past recombination events occurring in a set of germplasms, particularly landraces, and is performed by scanning genotype–phenotype associations along the chromosomes of the given germplasms. GWAS requires a large number of germplasms (several hundred to a few thousand) with high diversity and a large number of SNPs (several hundreds to tens of thousands). Compared to biparental mapping, GWAS provides higher QTL resolution, often to the gene level. Therefore, causative genes for a given trait can be identified by GWAS. In the past, GWAS could only be used for models and major crops where large numbers of SNP markers were available. With the advanced development of sequencing technologies, large numbers of SNPs in all crop species can now be discovered and genotyped by NGS, thus allowing the possibility of using GWAS in orphan crops like mungbean. The power of GWAS depends on the strength of correlation (the degree of LD) between the genotypes of markers and those of causative genes, which is a function of the distance between them, and the resolution of a QTL mapped by GWAS depends on how rapidly the LD decays over that distance (Myles et al., 2009). The LD extent is about 72–290 kb in cultivated mungbean (Noble et al., 2018; Ha et al., 2021; Sandhu and Singh, 2021) and 3–60 kb in wild mungbean (Noble et al., 2018).

GWAS for agronomic traits (PLH and DFL) and seed size (100 SDW) in a USDA mungbean collection of 482 accessions was carried out using 264,550 SNPs (Sandhu and Singh, 2021). Three SNP loci on different chromosomes were detected for each trait. For DFL, SNP 1_11367629 on chromosome 1 and 5_4604047 on chromosome 5 showed *R*^2^-values higher than 25%, while the other SNPs showed *R*^2^-values of about only 1%. Among the three loci for PLH, only SNPs 1_11367629, with *R*^2^ of about 30%, appeared to be correctly identified, while the others, with *R*^2^-values of 0%, were likely false positives. The major loci for 100 SDW were on chromosomes 1 and 7, and each QTL contributed 10–13% of the seed weight variation. Notably, SNP 1_11367629, located in *LOC106774729* and producing receptor-like protein kinase FERONIA (FER), was associated with all three traits. FER has diverse functions in plant growth and development, including hypocotyl and root elongation, root hair development, and flowering time (Deslauriers and Larsen, 2010; Duan et al., 2010; Wang et al., 2020; Zhu et al., 2020). We looked into an 83 kb region (Vr01:11309527..11393240) covering *LOC106774729* and found five other FER genes located next to *LOC106774729*. It will therefore be difficult to determine the causative FER genes for these traits. We also surveyed the region around SNP 5_4604047 and found that this marker is about 25 kb away from a *Phytochrome* gene. However, none of the DFL QTLs detected in this study were in the same region as *VrPHYA*, the candidate gene for DFL reported by Hwang et al. (2017). Nonetheless, the relationship between the seed weight QTLs identified by this GWAS and those detected by biparental mappings is not known.

The seed coat color of mungbean can be green, black, yellow, or brown. The color affects the consumer and industry preferences. Although seed coat color is a qualitative trait, several genetic models with up to five genes have been proposed for the inheritance of this trait in mungbean (reviewed in Dikshit et al., 2020). A GWAS of 209 cultivated and 9 wild mungbean accessions from 23 countries using 18,171 SNP markers detected a single SNP located on chromosome 4 in *Vradi04g08950* (positions 1,7682,006 to 17,685,676) encoding a pectinesterase associated with seed coat color variation determined by red, green, and blue (R, G, B) color components (Daovongdeuan, 2017). A GWAS for seed coat color variation in 466 cultivated accessions from various regions and 16 wild mungbean accessions using 22,230 SNPs revealed 9 SNPs located on four chromosomes associated with color variation (Noble et al., 2018). Five SNPs clustering and spanning a 22-kb region (positions 17,668,384 to 17,690,573) on chromosome 4 harbored a gene encoding a transcription factor, MYB113, known to be involved in anthocyanin biosynthesis, which is the candidate gene for black seed coat coloring, and an SNP on chromosome 5 was associated with a gene encoding flavonoid 3'-hydroxylase, which controls yellow seed color in soybean (Noble et al., 2018). The 22 kb region covered the SNP for seed color reported by Daovongdeuan (2017). In another study, GWAS was used to identify SNPs at positions 4,639,979 and 17,694,108 on chromosomes 2 and 4, respectively, associated with seed coat color (Sandhu and Singh, 2021). Anthocyanins are present in the seed coats of black mungbean seeds but absent in the seed coats of green, yellow, and brown seeds (Pandey et al., 1989). However, our exploration into the 22 kb region reported by Noble et al. (2018) found no MYB gene, but we did find *Vradi04g08920* (*VrMYB16*) encoding MYB16 and *LOC106758748* (*VrMYB90*) encoding MYB90, located about 38 and 10 kb from the region, respectively.

MYB16 and MYB90 are R2R3–MYB transcription factors that regulate various biological processes in land plants, including anthocyanin biosynthesis. Anthocyanins are pigments that give rise to red, purple, blue, and black colors in plants. MYB16 has been demonstrated to inhibit anthocyanin synthesis in apples (Xu et al., 2017), while MYB90 has been shown to regulate anthocyanin production in the tissues of several plant species (Qi et al., 2020), including mungbean (Lin et al., 2022). Map-based cloning, gene overexpression analysis, and haplotype analysis revealed that *VrMYB90* is responsible for anthocyanin synthesis in the mungbean hypocotyl, possibly through the upregulation of *VrDFR1*, encoding dihydroflavonol 4-reductase, at the last step of the synthesis, but the gene is not responsible for the black color of the seed coat (Lin et al., 2022). Thus, at this stage, it is reasonable to hypothesize that *VrMYB16* may control black seed coloring in mungbean. It is noteworthy that the germplasm used in the GWAS of Daovongdeuan (2017) and Noble et al. (2018) contained a small portion of black mungbean seeds (<5%). Most of the black mungbean in these studies were wild varieties. A strong population structure and genetic relatedness can prevent GWAS from correctly identifying causal variants and cause the identification of spurious associations. Moreover, in wild mungbean, the seeds are generally dull and brownish-black or black, and when the seed texture is removed, the green or mottled green-black color of the shiny seed is revealed (Watt et al., 1977; Sarikarin et al., 1999). Therefore, phenotyping seed coat colors must be performed with caution. Additional genomics studies should be conducted to find out the relationship between *VrMYB16* and seed coat color.

The GWAS of a small set of germplasms of 95 cultivated mungbean accessions (mostly from India and Pakistan) with 6,486 SNPs detected 43 SNP markers from 35 chromosome regions associated with calcium, iron, potassium, manganese, phosphorus, sulfur, and zinc concentrations in seeds (Wu et al., 2020). Some of the SNPs/regions were associated with more than one mineral, suggesting pleiotropic effects. In most cases, the significant SNP markers explained about 10–34% of the mineral variations. Twenty-six candidate genes related to various biological functions were identified for the seed mineral content, generally related to metal binding, metal translocation, and mineral uptake.

The GWAS for mungbean tolerance to biotic and abiotic stresses has also been carried out. GWAS for resistance to Fusarium wilt disease, caused by *Fusarium oxysporum*, was conducted in a USDA mungbean collection of 482 accessions, with 264,550 SNPs identified 3 loci associated with resistance (Sandhu and Singh, 2021). However, the highest *R*^2^ among these loci was only 6.8%. GWAS of phosphorus use efficiency in 144 mungbean accessions (mostly from India, along with Thailand, Taiwan, and Bhutan) with 55,634 SNPs identified 136 SNPs of 77 annotated genes of all 11 mungbean chromosomes associated with response to low-P and optimum-P concentrations under hydroponic conditions; 84 and 29 SNPs were related to root architecture and leaf area, respectively (Reddy et al., 2020). Based on the functions of candidate genes, 13 genes were chosen as candidate genes for the response and genes *Vradi01g05520, Vradi04g10750*, and *Vradi11g08340* were found to possess SNPs causing amino acid changes (Reddy et al., 2020). GWAS of salt stress tolerance (seed germination at 50 mM NaCl) of the minicore collection of WorldVeg mungbean with 5,288 SNPs (see details below) identified two genome regions on chromosomes 7 and 9 associated with tolerance (Breria et al., 2020). Although the QTL regions were very large (about 1 Mb), *Vradi07g01630*, encoding the ammonium transport protein, was selected as the candidate gene for QTL on chromosome 7, while *Vradi09g09510* and *Vradi09g09600*, encoding OsGrx_S16-glutaredoxin subgroup II and dnaJ domain proteins, respectively, were considered as candidate genes for QTL on chromosome 9 (Breria et al., 2020). Since salt stress tolerance is a complex trait and affects plants at all stages of growth and development, additional genomics studies should be conducted to elucidate the salt stress tolerance mechanism in mungbean.

In addition to the traits mentioned above, GWAS has been carried out for seed coat luster, hypocotyl color, pod color, and leaf drop at maturity (Sandhu and Singh, 2021).

## Mungbean Genetic Resources and Genome Diversity

Genetic resources are essential for breeding new crop cultivars and useful for gene mining *via* genomics research. Considerable genetic resources are available for mungbean. The largest collection of mungbean germplasm is maintained by the World Vegetable Center in Taiwan (10,438 accessions; https://genebank.worldveg.org). Mungbean germplasm collections with large numbers of accessions are also conserved in the national genebanks of India (11,000 accession) (Gayacharan et al., 2020), USA (3,931 accessions; www.genesys-pgr.org), the Philippines (1,623 accession), Australia (1,385 accessions; www.genesys-pgr.org), Japan (1,455 accessions; www.gene.affrc.go.jp), Korea (1,091 accessions; http://genebank.rda.go.kr), Russia (877 accessions; www.genesys-pgr.org), and the United Arab Emirates (408 accessions; www.genesys-pgr.org). Several thousand mungbean accessions are also conserved in the national genebank of China, although the exact number is not known. The mungbean collection in Russia is the oldest, with most of the accessions collected between 1910 and 1927 (Gayacharan et al., 2020), while the collection maintained in Japan has the largest number of wild mungbean, 211 accessions (www.gene.affrc.go.jp). The availability of these large collections of mungbean germplasm provides opportunities to discover new, beneficial genes and study their functions for breeding through genomics analysis.

There are only a few reports on the molecular genetic diversity of large collections of mungbean. A collection of 615 mungbean accessions (415 cultivated, 189 wild, and 11 weedy) covering all distribution areas were analyzed with 19 azuki bean SSR markers (Sangiri et al., 2007). The number of alleles found in wild mungbean (257) was about double that found in cultivated mungbean (138), while the gene diversity (*H*_E_) in cultivated mungbean was 65.01% of that in wild mungbean. For cultivated mungbean, the greatest diversity was found in India and Pakistan. For wild mungbean, the highest diversity was found in South Asia (*H*_E_ = 0.68), but the level of diversity was not much different from that in Australia (*H*_E_ = 0.59). These results support the hypotheses of Tomooka et al. (1992). However, the Australian wild mungbean is distinctly different from Asian and African wild mungbean at both the genetic and phenotypic levels (Lawn and Rebetzke, 2006; Sangiri et al., 2007; Ha et al., 2021); thus, the wild mungbean can be used to enrich the gene pool of cultivated mungbean in breeding.

A core collection comprising 52 cultivated and 49 wild accessions was developed based on SSR profiles and phenotypic data (Sangiri et al., 2007). In another study, 705 cultivated mungbean accessions from 26 countries (about 60% from Korea) were assessed for diversity using 695 AFLP and 15 SSR markers (Moe et al., 2012). The gene diversity of the germplasm estimated by AFLP and SSR markers was 0.69 and 0.36, respectively. A core collection of 222 accessions was constructed based on AFLP markers (Moe et al., 2012). A core collection of 1,481 mungbean accessions was developed from 5,234 accessions of cultivated mungbean maintained at WorldVeg based on geographical information and eight agronomic traits (Schafleitner et al., 2015). In addition, this core collection was molecularly assessed using 20 SSR markers and, as a result, a minicore collection of 297 accessions was developed. Genetic diversity in the core and minicore collections was moderate, with 122 and 166 alleles and *H*_E_ of 0.49 and 0.57, respectively. This minicore collection was further characterized using 5,288 SNP markers generated by genotyping-by-sequencing (GBS), revealing that the collection is composed of four genetic clusters that are generally related to the geographical origins of the mungbean. In general, mungbean from South Asia (India and Pakistan) were clustered with accessions from West Asia, but separated into different subclusters, while those from other regions were subdivided and clustered together.

The core/minicore collections developed by Sangiri et al. (2007) and Schafleitner et al. (2015) are useful resources for QTL and gene identification by GWAS and are available to the research community. Combining the core collection developed by Sangiri et al. (2007) and the minicore collection reported by Schafleitner et al. (2015) can enhance genome diversity, utilization of genetic resources in mungbean genomics research, and genetic improvement. Recently, a set of mungbean germplasms with 276 accessions (233 cultivated and 42 wild) from 23 countries was assessed for diversity using genome-wide SNP variations analyzed by GBS (Ha et al., 2021). The assessment showed that the average nucleotide diversity in cultivated mungbean was more than 3-fold lower than that in wild mungbean, and the cultivated mungbean from India showed the closest relationship with the wild mungbean. Hence, it is clear that cultivated mungbean have lost much of their genetic diversity through domestication and breeding. Wild mungbean germplasm is an important source of resistance to biotic and abiotic stresses (Fujii and Miyazaki, 1987; Lawn et al., 1988; Lawn and Rebetzke, 2006), which have become more prominent and serious owing to climate change. The genomes of wild mungbean should be explored by WGS and whole-genome resequencing to harness their natural allelic variation for breeding climate-resilient mungbean.

## Marker-Assisted Breeding

Conventional and molecular breeding are the two approaches used to develop crop cultivars. Genomics research provides tools (such as DNA markers), informatics, and knowledge that can enhance the efficiency and precision of conventional plant breeding. The application of DNA markers for decision-making in plant selection processes is called marker-assisted breeding (MAB), which is a kind of molecular breeding (Collard and Mackill, 2008). MAB is composed of marker-assisted selection (MAS) and genomic selection (GS). MAS utilizes markers that are closely or tightly linked with traits of interest to select plants with desirable allele-affecting target traits (Collard and Mackill, 2008), while GS employs genome-wide markers to predict the effects of all loci and thereby calculate genomic estimated breeding values for all individuals of a breeding population (Goddard, 2009).

Simple sequence repeat markers are widely used in MAS because they are readily available and comparatively cheaper than others, and they require a simple technique with a higher polymorphism rate. MAS has been successfully applied in several major crops, but rarely in minor and orphan crops, including mungbean. As a result of limited progress in mungbean genomics research, only a few simple and efficient markers closely linked to phenotypic traits have been reported and validated recently, as mentioned above; thus, few MASs have been reported in mungbean. Marker-assisted backcrossing (MABC) was practiced to introduce *VrPGIP2* from V2802 and *VrMLO12* from V4718 to mungbean cultivar “Chai Nat 84-1” to improve resistance to bruchids and powdery mildew, respectively (Jittawimon, 2021). Papan et al. (2021) enhanced the CLS and PM resistance of mungbean cultivar “KING” *via* MABC using *qCLSC72V18-1* (*VrTAF5*?) from V4718 CLS, and *qPMC72V18-1* from V4718 and *qPMC72V85* from V4785 for PM. Wu et al. (2022) improved bruchid resistance in the mungbean cultivar “Kamphaeng Saen 1” using *VrPGIP2* from V2802 by MABC.

## Conclusion

Since the publication of the first genomic mapping of mungbean in 1992, mungbean has entered the genomics era. Unfortunately, like other minor or orphan crops, mungbean has received little attention in the research community over the past 30 years; thus, it has not been established as a model crop species for genomics research. Despite the slow progress in genome research due to the limited genomic resources, the last 10 years have witnessed an increase in genomic studies in mungbean, resulting in significant findings and knowledge regarding some complex and important traits, especially insect and disease resistance, accumulated, thanks to advanced sequencing technologies and aiding in the construction of reference genome sequences of mungbean and genotyping of SNP markers.

The majority of mungbean genomics research is concentrated on resistance to a few diseases (PM, CLS, and YMD) and insect pests (bruchids and bean bug), while research on other traits, such as resistance to root diseases, tolerance to environmental stresses (e.g., scarce moisture, excessive moisture (waterlogging and preharvest sprouting), and high temperature), and the nutrient and chemical composition of seeds, has not been done or is very rare. At present, several omics areas, such as transcriptomics, proteomics, and phenomics technologies, have advanced, but only a few have been integrated into mungbean genomics research. Applying omics methods could accelerate and strengthen genomics research to solve the complexity of these traits in mungbean.

QTL mapping and information are the basis for MAB and GAB. Only biparental mapping populations have been used in mungbean. Generally, biparental populations provide low-resolution and imprecise QTLs of complex traits. Populations derived from intercrossing multiple parents, including nested association mapping (NAM) (McMullen et al., 2009) and multiparent advanced generation intercross (MAGIC) (Cavanagh et al., 2008) populations, have been proposed for use in high-resolution, precise QTL mapping. To date, two NAM mungbean populations (Nair et al., 2022) and one MAGIC mungbean population (Somta, unpublished data) have been developed, which will improve efficiency and precision in the genetic dissection of complex traits in mungbean.

## Future Perspectives

In recent years, mungbean has been gaining interest from consumers and food industries and growing in popularity globally due to its high nutritional density, leading to increased attention being paid to the development of new mungbean cultivars that meet the demands of farmers, consumers, and industries in different countries by exploiting natural genetic variation through genomics. This is reflected by a considerable number of publications on the use of genomics and other omics sciences in mungbean from several laboratories within the last 5 years. Although advanced sequencing technology has revolutionized mungbean genome research, the genomics resources (such as high-quality genome sequences) still lag behind other crops, and genome information (such as QTLs) is still limited to a few traits and is insufficient for MAB and GAB. In the future, therefore, the main tasks in mungbean genome research will be to (i) construct complete, high-quality chromosome-scale reference genomes; (ii) identify the QTLs and genes controlling agronomically important traits, yield, quality, and resistance to biotic and abiotic stresses, as well as the molecular mechanisms underlying these traits; (iii) unravel genome variation in diverse mungbean germplasms (pan-genome analysis and assembly); (iv) create comprehensive databases of mungbean genomes and resources with the ability to analyze the genomic data; and (v) develop a highly efficient regeneration protocol and transformation system to support functional genomics. In addition, besides GSB, other SNP genotyping platforms, such as kompetitive allele-specific PCR (KASP) assay and SNP array, should be developed for mungbean to facilitate MAB and GAB.

To date, genomes of several other cultivated and wild *Vigna* species have been sequenced and are publicly available, and genomes of these species are highly syntenic with mungbean at both the micro and macro scale (Sakai et al., 2015; Lonardi et al., 2019; Pootakham et al., 2021; Guan et al. submitted) https://viggs.dna.affrc.go.jp). These *Vigna* genome sequences are valuable resources for gene identification and functional gene analysis of mungbean *via* comparative genome analysis. Since mungbean is a minor crop with a small research community and limited research funds, we propose that an international consortium for mungbean genomics research, making a collaborative effort to produce a body of genetic and genomic resources, should be established to accelerate mungbean genome research. Recently, the International Mungbean Improvement Network (IMIN), with the aim of strengthening local mungbean research, pre-breeding, and variety development capacity to generate improved, farmer-accepted varieties, was established (Nair et al., 2022). The IMIN is led by the WorldVeg with partners from Australia, Bangladesh, India, Myanmar, Kenya, Tanzania, and Uganda, and has generated resources and knowledge on mungbean genomics.

## Author Contributions

PS conceived the idea and designed the structure of this manuscript. PS and KL revised the manuscript. All authors collected literature, wrote the manuscript, contributed to the article, and approved the submitted version.

## Funding

This work was supported by the National Research Council of Thailand (NRCT) (Grant No. N42A650274), Kasetsart University Research and Development Institute, the National Key R&D Program of China (2019YFE0109100), the China Agriculture Research System of MOF and MARA-Food Legumes (CARS-08-G15), and the Jiangsu Seed Industry Revitalization Project [JBGS(2021)004].

## Conflict of Interest

The authors declare that the research was conducted in the absence of any commercial or financial relationships that could be construed as a potential conflict of interest.

## Publisher's Note

All claims expressed in this article are solely those of the authors and do not necessarily represent those of their affiliated organizations, or those of the publisher, the editors and the reviewers. Any product that may be evaluated in this article, or claim that may be made by its manufacturer, is not guaranteed or endorsed by the publisher.
