# Supplementary material for: Thirty Years of Mungbean Genome Research: Where Do We Stand and What Have We Learned?
Source: Front Plant Sci. 2022 Jul 15;13:944721. doi: 10.3389/fpls.2022.944721 (PMC9335052; doi:10.3389/fpls.2022.944721)

Supplementary Figure S1. Location of the QTL *Sdwa5.3.1+* on LG3 and possible gene (*LOC106764165*) controlling seed dormancy detected in BC_1F_1 population of a between a wild mungbean 'JP211874' and cultivated mungbean 'JP229096' ('Sukhothai'). The QTL was re-analyzed by inclusive composite interval mapping method using original marker genotypes and phenotype reported by Isemura et al. (2012), but removing a cluster of markers detecting more than one locus around the original QTL location. Not all the original markers were include in the QTL mapping. *LOC106764165* encodes gibberellin 2-beta-dioxygenase 1 (GA2OX1).

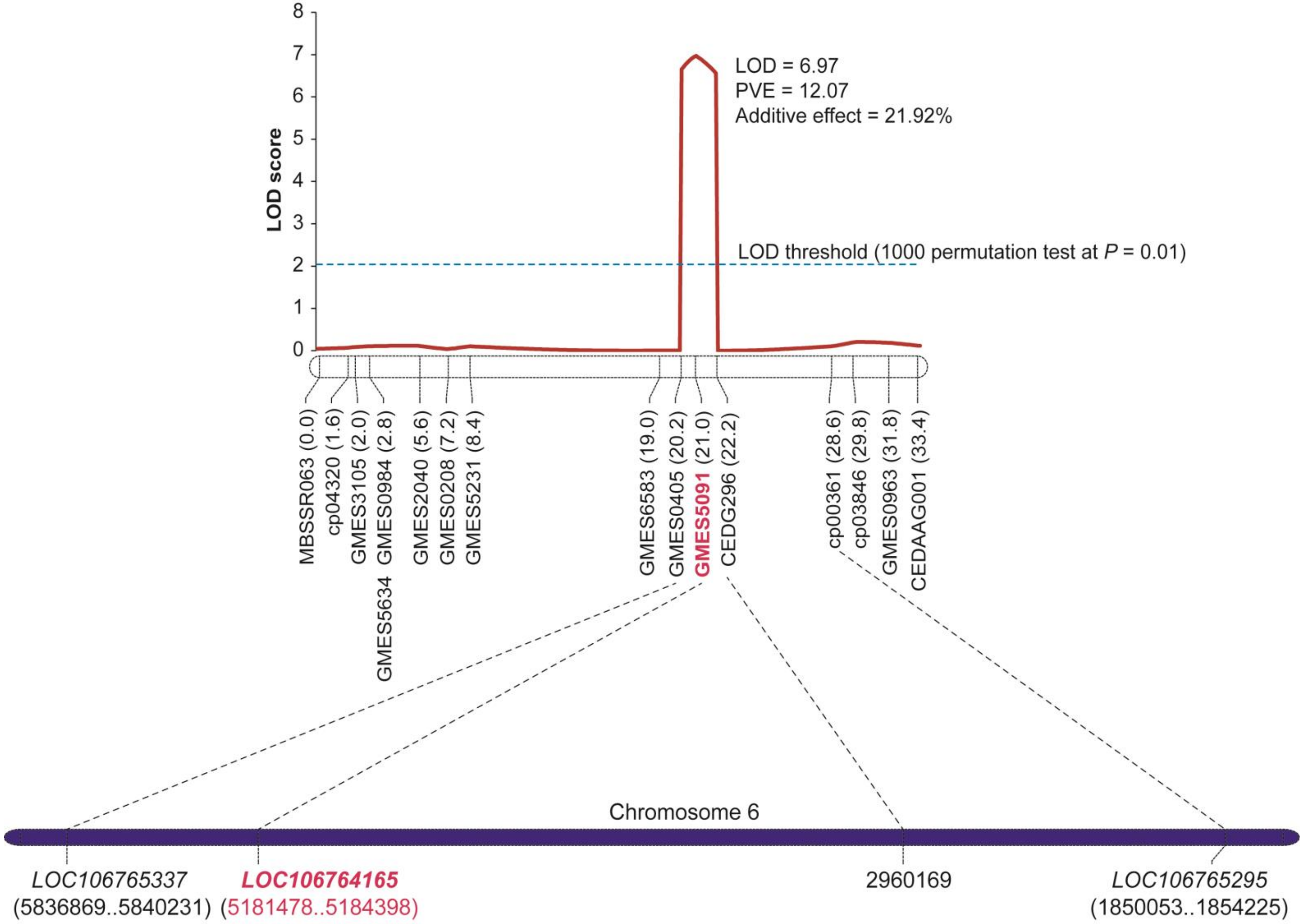

Supplement: Supplementary file 1 [file Image_1.pdf]
